# Activating the Right Hemisphere Through Left-Hand Muscle Contraction Improves Novel Metaphor Comprehension

**DOI:** 10.3389/fpsyg.2021.729814

**Published:** 2021-10-14

**Authors:** Tala Noufi, Maor Zeev-Wolf

**Affiliations:** ^1^Department of Education, Ben-Gurion University of the Negev, Beersheba, Israel; ^2^Zlotowski Center for Neuroscience, Ben-Gurion University of the Negev, Beersheba, Israel

**Keywords:** figurative language, novel metaphors, lateralization, cognitive enhancement, right hemisphere

## Abstract

The neurotypical brain is characterized by left hemisphere lateralization for most language processing. However, the right hemisphere plays a crucial part when it is required to bring together seemingly unrelated concepts into meaningful expressions, such as in the case of novel metaphors (unfamiliar figurative expressions). The aim of the current study was to test whether it is possible to enhance novel metaphor comprehension through an easy, efficient, and non-invasive method – intentional contraction of the left hand’s muscles, to activate the motor and sensory areas in the contralateral hemisphere. One hundred eighteen neurotypical participants were asked to perform a semantic judgment task involving two-word expressions of four types: literal, conventional metaphors, novel metaphors, or unrelated, while squeezing a rubber ball with their right hand, left hand, or not at all. Results demonstrated that left-hand contraction improved novel metaphor comprehension, as participants were more accurate and quicker in judging them to be meaningful. The findings of the present work provide a simple and efficient method for boosting right hemisphere activation, which can be used to improve metaphoric language comprehension. This method can aid several populations in which right hemisphere function is not fully established, and who struggle with processing figurative language, such as adolescents and individuals on the autistic spectrum.

## Introduction

It has long been established that there is a left-hemisphere (LH) bias in most language-related functions (Beeman, 1993, 2005; Fiore and Schooler, 1998; Josse and Tzourio-Mazoyer, 2004). When exposed to a word, the LH activates broad semantic associations; however, it quickly suppresses and constrains any associations that are subordinate or irrelevant to the context, or remote. This characteristic of the LH is called *fine-semantic coding* (Beeman et al., 1994). Although the LH is sufficient for most basic language processing, the right hemisphere (RH) also plays an important role in comprehending language (Beeman et al., 1994). While the LH strongly stimulates a more focused semantic field, the RH maintains (for a longer period) a broader, more subtle, and weaker semantic field (*coarse semantic coding* – Beeman, 2005). Beeman’s studies indicate that by activating loose, expanded, and dull associations of input words, the semantic-processing mechanisms in the RH help individuals to process complex language tasks, which include deriving secondary and unconventional meanings of words (Beeman et al., 1994; Beeman, 2005). It has been further suggested that this special semantic processing in the RH is what allows the comprehension of natural language beyond literal meaning – i.e., figurative language (Beeman et al., 1994; Beeman, 2005; Faust, 2012; Zeev-Wolf et al., 2014; Patael et al., 2018).

Figurative language plays a substantial part in our lives (Gibbs et al., 2002). Many expressions in daily communication rely on these word combinations, which add richness, color, and interest to conversation (MacKay and Shaw, 2004). Take, for example, the expression *“It’s raining cats and dogs”*: while it is impossible for the sky to literally rain animals, it is generally understood that the meaning behind this expression is heavy rain. To convert such word combinations into meaningful expressions, the RH must be involved, as it is necessary to create associative links between concepts with distant meanings (Beeman, 2005). The creation of remote associative links in the brain relies on the stimulation of a wider range of associations to a given word, or concept, that occurs in the RH (Beeman et al., 1994; Beeman, 2005; Faust, 2012; Zeev-Wolf et al., 2014; Patael et al., 2018). However, the processing of figurative language requires not only the RH, but a balanced exchange between the two hemispheres (Beeman, 2005), as the LH also plays the role of a gatekeeper – suppressing all unrelated, irrelevant, associations that may arise in the RH (Pobric et al., 2008). Through the RH and LH’s interaction, the brain establishes the ability to distinguish between meaningful and meaningless words and phrases, and thereby to comprehend figurative language.

The RH contribution to and involvement in language processing is supported by evidence from multiple sources. For instance, the *split visual field paradigm* – a behavioral method used to test the various functions of each hemisphere in fine-coarse semantic coding, by presenting a stimulus either to the left visual field (which is connected directly to the RH) or the right visual field (which is connected directly to the LH) – has demonstrated hemispherical differences in language processing (Beeman, 2005; Faust and Mashal, 2007; Zeev-Wolf et al., 2014). Semantic relations are processed differently, depending on whether they were presented to the left visual field, or the right one (Beeman, 2005). For example, in their study, Faust and Mashal (2007) showed that novel metaphors (NM) – unfamiliar figurative expressions – were processed faster and more accurately when target words were presented to the RH (via the left visual field). Another example of RH contribution is found in neuroimaging studies revealing neural activity in the RH (albeit weaker than in the LH, in the intact brain – Bookheimer, 2002; Beeman, 2005; Démonet et al., 2005; Xu et al., 2005). In addition, several studies have indicated that healthy subjects show greater RH activation (compared with the LH) while performing higher language tasks – such as (inter alia) comprehending figurative language (Bottini et al., 1994; Sotillo et al., 2004; Coulson and Wu, 2005; Mashal et al., 2005, 2007). Further support for this theory comes from Beeman et al. (1994), who found that when people with RH damage try to comprehend figurative language, they manage to do so only in a limited and literal manner. Another example of hemispheric dysfunction comes from schizophrenia patients, who, some studies suggest, have reduced language lateralization – showing a more bilateral pattern (Crow, 1997; Sommer et al., 2001; Tréhout et al., 2017), resulting in over-activation of the RH to language – which leads them to overuse coarse semantic coding, even when it is not needed – but gives them an advantage in grasping novel metaphors (Zeev-Wolf et al., 2014, 2015).

Novel metaphors are a unique type of figurative language. Unlike conventional metaphors (CM), they are entirely new and unfamiliar expressions – meaning that comprehending and processing them requires the ability to put together and make sense of new and (seemingly) meaningless expressions. In other words, the RH’s coarse-semantic coding is called for when encountering NM. Conversely, CM are familiar metaphors, that are frequently used in our daily conversations (Giora, 2007) – so much so that they eventually become “dead” metaphors, whose comprehension requires fewer cognitive and linguistics abilities than NM (Giora et al., 2000; Giora, 2007). Put differently, the processing of CM is based on fine, rather than coarse, semantic coding – as has been previously shown (Zeev-Wolf et al., 2014).

Interestingly, the fine balance between the hemispheres is flexible. It has been shown that the performance of each hemisphere may, in fact, be intentionally activated, suppressed, or manipulated – resulting in changes to the fine/coarse semantic coding balance (Pobric et al., 2008; Goldstein et al., 2010). For example, a study by Pobric et al. (2008) – aimed at examining metaphor comprehension, while suppressing the function of the LH and RH using repetitive transcranial magnetic stimulation – found that suppression of brain regions related to semantic processing in the RH disrupted processing of NM, while suppression of brain regions related to semantic processing in the LH disrupted processing of literal expressions (LIT) and CM.

Other examples of hemispheric manipulation have been demonstrated in several studies, that suggest that an intentional muscle contraction of the muscles of one’s hand (or one’s face) can boost the performance and involvement of the motor and sensory areas of the contralateral hemisphere (Schiff et al., 1998; Bassel and Schiff, 2001; Goldstein et al., 2010). According to Christie et al. (2008), exercise has been proved to make alterations in the cerebral vasculature which affect physiological changes in the brain such as learning and memory. It is probable then, that boosting blood flow to the brain (e.g., to the RH) through some type of physiological exercise (e.g., left-hand contraction) might increase neural activity (e.g., in the RH) and thus improve language processing and comprehension. It is important to note that, according to some neuroimaging and neurophysiological studies, motor production areas are involved in speech perception and language production tasks (Gunter et al., 2002; Meister et al., 2007; Londei et al., 2010). That is, the motor and language areas in the brain are not autonomous and independent but rather connected. Thus, hand contractions that result in activation of the neural activity in the contralateral hemisphere through blood flow, activate motor areas which are also related to language (Pulvermüller, 2005); Moreover, from an anatomical perspective, increased blood flow to motor and sensory brain areas due to physical activity can be utilized by proximate areas if needed. That is, motor cortex activation might spread to other regions through cortico-cortical connections (Pulvermüller, 2005).

Studies show that unilateral hand-contractions (leading to spread of activation from the motor and sensory areas to other proximate areas of the brain) can have significant, and divergent, emotional, behavioral, and cognitive impacts, depending on which hand is the source of the activity (Schiff and Lamon, 1994; Harmon-Jones, 2006; Goldstein et al., 2010). For example, left-hand contractions have been found to trigger sadness, and a tendency to have negative judgments and perceptions (Schiff and Lamon, 1994; Harmon-Jones, 2006). Moreover, right-hand contraction – which leads to LH activation, reduced the negative emotional tones and attitudes among individuals completing the Thematic Apperception Test, and increased individuals’ determination when encountered with intractable problems (Schiff and Lamon, 1994; Goldstein et al., 2010). In addition, the possibility of cognitive consequences of divergent thinking through unilateral hand-squeezing was also tested in a study by Goldstein et al. (2010). In it, individuals had to squeeze a ball with either their left or their right hand, and then complete a creativity test. Creativity – which is mainly related to the performance of the RH (Goldstein et al., 2010), as it depends on the ability to bring together seemingly unrelated concepts – improved among individuals that had squeezed the ball with their left hand, thereby boosting the performance of their RH. The study also showed that left-hand contractions did, in fact, result in greater cognitive value, enhanced effects, and RH activation than right-hand contractions. That said, a replication study (albeit in different settings) did not find the same effects (Turner et al., 2017).

Although Turner et al. (2017) study supported the notion that unilateral hand-contractions can be used in cognitive experiments to boost the activation of the contralateral hemisphere, their results showed that the LH, rather than the RH, enhanced participants Remote Associates Test (RAT) scores. They postulate that these findings were mainly a result of the language used in the task. While Goldstein et al. (2010) used Hebrew, Turner et al. (2017) used English. They postulate that while Hebrew RAT problems can be solved using insight (i.e., relying more on the RH), English RAT problems can only be solved analytically (i.e., relying more on the LH). Moreover, Turner et al. (2017) suggest that even though previous studies have shown the effectiveness of unilateral hand-contractions on emotion, motivation, and cognition, its usefulness is probably limited to specific tasks.

Another study done by Stanković and Nešić (2020) used photographs to investigate the effect of unilateral hand-contractions on the perception of emotions; however, no significant results were found. One limitation which may have contributed to this result is the small sample size used. Additionally, the unilateral hand-contractions method, used in manipulating and assessing the hemispheric balance, was followed by the presentation of pictures to either the left or the right visual field. That is, in addition to the physiological manipulation, there was a visual manipulation that might have altered the results.

Nonetheless, based on the above, we hypothesized that unilateral muscle contractions of the left-hand (by squeezing a ball) would improve NM processing (i.e., by boosting RH coarse semantic processing, participants would respond more accurately, and quickly, to NM) compared with participants who squeezed the ball with their right hand, or Control participants (who did not squeeze a ball at all) – but would not improve the processing of CM, literal expressions (LIT), or unrelated expressions (UR), which rely on LH processing (fine semantic coding).

## Materials and Methods

### Participants

Sample size was calculated using G^∗^power computer software for power analysis (Erdfelder et al., 1996). The analysis indicated that a sample of 120 participants would be sufficient to detect a small to medium effect size (*f* = 0.15) with 95% power. A hundred and twenty students over the age of 18 from Ben-Gurion University of the Negev were recruited for the study, in return for course credit, or cash, for taking part. All those taking part in the research were right-handed (assessed using the Edinburgh Inventory; Oldfield, 1971); native Hebrew speakers with normal eyesight; no history of head injuries or neurological disorders; and no reading impairments (assessed using the Psycholinguistic Assessments of Language Processing in Aphasia Tool; Kay et al., 1992). In addition, their verbal intelligence was assessed by means of the Similarities Subtest of the Hebrew version of the Wechsler Adult Intelligence Scale, to rule out differences in verbal intelligence between the groups (Wechsler, 1997). Two participants were excluded from the analysis, due to a deviant pattern of results (no correct responses to one or more of the conditions) – resulting in a final sample of 118 participants. The research was conducted after obtaining institutional approval from the University’s Human Subjects Research Committee, and in accordance with the academic ethical code. For demographic details, see Table 1.

**TABLE 1:** Demographic details.

	Right-hand (*n* = 39)		Left-hand (*n* = 39)		Control (*n* = 40)		Analysis	
	Mean	SD	Mean	SD	Mean	SD	*F* (df = 2)	*p*
Age (years)	25.2	2.6	24.8	2.1	24.45	2.4	0.87	0.42
Education (years)	13.77	1.4	13.67	0.11	13. 48	0.99	0.64	0.52
Handedness	95.07	7.2	96.43	6.06	96.92	5.28	0.92	0.40
Similarities (standard score)	10.1	1.38	10.3	1.01	10.2	0.97	0.30	0.74
PALPA test score	59.5	0.71	59.7	0.69	59.48	0.71	1.19	0.31

	**N**	**%**	**N**	**%**	**N**	**%**	***χ*^2^ (df = 2)**	***p***

Male	11	9.3	11	9.3	11	9.3	0.01	0.99
Female	28	23.72	28	23.72	29	24.58		

### Stimuli

The stimuli pool used for this study was taken from previous research (for more information, see Gold and Faust, 2010), and comprises 240 Hebrew prime-target word pairs. The 240-word pairs formed four types of semantic expressions (60 for each category): LIT (e.g., *soft blanket*); CM (e.g., *juicy gossip*); NM (e.g., *wilting hope*); and unrelated word pairs that form meaningless expressions UR (e.g., *picturesque concern*). The use of word pairs, which are considered a single unit, is commonly used in Hebrew and is a popular form of language usage in spoken and written language due to its economy and elegance. For more examples, see Table 2. The translation of the word pairs is reversed (as opposed to English), and that is due to the Hebrew grammar. All prime words are nouns, and all prime and target words consisted of two to six letters. Word length was counterbalanced across the four types of word-pairs (each condition contained equal numbers of 2, 3, 4, 5, and 6 letter primes and targets). Stimuli were also balanced between conditions according to word frequency, concreteness, grammatical category, and syntactic structure. Gold and Faust (2010), performed multiple pretests in order to determine the type of semantic relationship, concreteness and word frequency between the words in each pair. Moreover, as detailed in previous studies, considerable pre-testing by 40 judges (who did not take part in the experiments), was carried out, to decide whether the two-word expressions were literally plausible, metaphorically plausible, or implausible. Only the novel expressions that were rated by at least 80% of the judges as metaphorically meaningful (for the NM category), or meaningless (for the UR category), were included in the study (Gold and Faust, 2010). It should further be noted that in the present study, the semantic expressions were presented in the absence of context, providing a purely neurolinguistic perspective.

**TABLE 2:** Examples of the four types of expressions and their English translation.

LIT		CM		NM		UR	
שרשרת פנינים	Pearl necklace	מלבב חיוך	Blossoming smile	מילים יצוקות	Firm words	נמר כינור	Violin tiger
בטון מערבל	Cement mixer	שפתיים חתומות	Sealed lips	גועש חלום	Stormy dream	חרם דלי	Ban bucket
קן נמלים	Ant nest	גורפת החלטה	Sweeping decision	עופרת גשמי	Leaden rain	דוד מדיני	State uncle

*LIT, literal expressions; CM, conventional metaphors; NM, novel metaphors; UR, unrelated expressions.*

### Procedure

Participants were tested individually in a quiet room, in the presence of the researcher, who documented any unusual behaviors. They were told that the goal of the study was to examine the relationship between motor and cognitive activities. After signing the informed consent form, and responding to the questionnaires (gauging handedness, reading impairments, and verbal intelligence), participants were randomly assigned to either the Control (no contractions), Left-Hand, or Right-Hand contraction conditions. Participants in the contraction conditions (either Left- or Right-Hand), were asked to squeeze a 7-cm-diameter rubber ball as hard as they could, while placing their other hand flat on the table. They were asked to do so for 4 min – alternately squeezing the ball for 45 s, then relaxing for 15 s, four times. In the control group, participants rested both hands with their palms facing the table, and waited 4 min in a relaxed state, before starting the experiment. This procedure was repeated three times throughout the experiment – once before the experiment began, and twice during the breaks given to the participants during the task, which were planned in advance.

Participants then sat facing a screen, with their head placed on a chinrest situated at a distance of 60 cm, and were asked to focus on the fixation sign (“+”) at the center of the screen, without shifting their gaze elsewhere. The word pairs were then presented in random order, with the first word (of each pair) presented for 650 ms – followed by a fixation sign (“+”) for 100 ms, and then the target word, for 180 ms. Participants were instructed to read the word pairs silently, and to decide whether they were meaningful expressions or not, by pressing either the right or left key with their index finger, as rapidly and as accurately as possible. Once they did so, and before the next words pair emerged, another fixation sign appeared at the center of the screen for 1,500 ms. All stimuli were presented in the center of the screen.

It should be noted that every participant was first given an exercise session involving 20 expressions, which was not included in the analysis. Moreover, they were told that some of the word pairs and expressions were figurative and were given illustrative examples (such as *mercy blanket*). They were also told that some expressions might be unfamiliar, yet still meaningful.

*Line bisection test.* Two line-bisection trials (LBT) were performed directly after the first hand-contraction priming paradigm, and at the end of the experiment (four trials in total), in a bid to check the certainty of hemispheric activation (i.e., the manipulation). Specifically, participants were presented with 180-mm black line printed horizontally across the middle of a white sheet of paper. On each attempt, participants were asked to thoroughly mark the precise center point of the line. In two of the trials, the line was printed closer to the right border of the page, and closer to the left border in the remaining two trials. Using a fine-point pen, participants were asked to bisect the line as accurately as possible. The scores reflect the deviation in mm from the actual center of the line: positive scores reflect a bias to the right side as a result of a stronger LH activation, and negative scores reflect a bias to the left side, as a result of a stronger RH activation. For each participant, an average score across the four attempts, was calculated (LB score).

## Results

Trials in which reaction time (RT) was longer or shorter than three standard deviations, for each condition for each participant were excluded from the analysis. The overall accuracy (AC) was 77.291% (SD = 6.836). AC did not covary across conditions with RT, *r* = −0.128, *p* = 0.167. Thus, no indication for trade off between response speed and accuracy was found. For mean AC and RT see Tables 3, 4. Statistical analyses were performed in R (R Core Team, 2021).

**TABLE 3:** Accuracy rate (%).

Type	Condition	Mean	SD
**LIT**	Control	92.92	3.62
	Right-Hand	93.72	3.39
	Left-Hand	94.78	3.6
**CM**	Control	87.8	7.56
	Right-Hand	86.5	8.08
	Left-Hand	92.98	5.7
**NM**	Control	27.21	19.64
	Right-Hand	26.82	23.32
	Left-Hand	58.96	24.7
**UR**	Control	92.01	7.29
	Right-Hand	90.32	11.09
	Left-Hand	88.7	11.2

*LIT, literal expressions; CM, conventional metaphors; NM, novel metaphors; UR, unrelated expressions.*

**TABLE 4:** Reaction time (ms).

Type	Condition	Mean	SD
**LIT**	Control	526.05	90.24
	Right-Hand	537.82	93.35
	Left-Hand	501.57	87.37
**CM**	Control	575.61	106.28
	Right-Hand	581.74	110.27
	Left-Hand	535.69	103.55
**NM**	Control	804.64	217.51
	Right-Hand	809.23	247.97
	Left-Hand	732.99	211.71
**UR**	Control	667.81	145.66
	Right-Hand	660.19	120.99
	Left-Hand	669.71	177.44

*LIT, literal expressions; CM, conventional metaphors; NM, novel metaphors; UR, unrelated expressions.*

### Line Bisection Test

One-way ANOVA, with hand as the between-subject variable, revealed a significant main effect: *F*(2,115) = 4.042, *p* = 0.02, η^2^ = 0.066. A pairwise comparison between conditions (using the error term from the original ANOVA) revealed that participants in the Left-Hand condition had smaller and negative scores (*M* = −0.853, SD = 4.746) – indicating a bias to the left as a result of RH activation – than participants in the Control (*M* = 1.236, SD = 4.041, *p* = 0.031, *d* = 0.475) or Right-Hand condition (*M* = 1.729, SD = 3.957, *p* = 0.009, *d* = 0.592), which yielded positive scores – indicating a bias to the right as a result of LH activation. No significant difference was found between participants in the Control condition and those in the Right-Hand condition (*p* = 0.608).

### Accuracy

Single trial accuracies were modeled with a generalized linear mixed model with a binomial likelihood function and a logit link function (a logistic regression analysis). The unconditional interclass correlation (ICC) was estimated separately for Participants and Items. A small yet significant amount of variation was found between participants in their overall accuracies (*N* = 119; *ICC* = 0.042, χ^2^(1) = 408.939, *p* < 0.001), and a large amount of variation was found between items (*N* = 240; *ICC* = 0.569, χ^2^(1) = 10,397.485, *p* < 0.001), indicating that, as expected, some items tended to evoke lower or higher rates of correct responses.

For the main analysis, hand (left, right, no-contraction) and expression-type (LIT, CM, NM or UR) and their interaction were modeled as fixed effects, with crossed random intercepts per participant and item. Due to convergence issues, we were unable to fully account for any possible random slopes nested within participants or item. We report here type 3 omnibus tests for the fixed effects.

The generic mixed-level equation, with all effects considered to be random, was:


$${\text{AC}}_{i}∼\text{Bernoulli}\left(Pr\left(\text{AC}=1\right)=\hat{P}\right)$$

$$\begin{array}{c} \text{log}\left[\frac{\hat{P}}{1-\hat{P}}\right]=\alpha_{\text{j}\left[\text{i}\right],k\left[\text{i}\right]}+\beta_{\text{c}}\times \mathrm{Expression}{\mathrm{Type}}_{\text{c}}+\beta_{\text{g}}\times {\text{Hand}}_{\text{g}}\\ +\beta_{\text{cg}}\times \mathrm{Expression}{\mathrm{Type}}_{\text{c}}\times {\text{Hand}}_{\text{g}} \end{array}$$

$$\alpha_{j}∼N\left(\mu_{\alpha_{j}},\sigma_{\alpha_{j}}^{2}\right),\mathrm{for}\mathrm{Item}j=1,\ldots ,J$$

$$\alpha_{k}∼N\left(\mu_{\alpha_{k}},\sigma_{\alpha_{k}}^{2}\right),\mathrm{for}\mathrm{Participant}k=1,\ldots ,k$$

The analysis revealed a significant main effect for both hand and expression-type conditions: χ^2^(2) = 11.791, *p* = 0.003, for hand condition, χ^2^(3) = 388.171, *p* < 0.001, for expression-type. A pairwise comparison with Tukey’s correction between conditions revealed that participants in the left-hand condition (*prob* = 0.922) were more accurate than those in the control condition (*prob* = 0.889; *OR* = 1.477, *z* = 2.67, *p* = 0.021) or right-hand condition (*prob* = 0.88; *OR* = 1.608, *z* = 3.219, *p* = 0.004). No difference between control and right-hand condition was found (*p* = 0.825). Pairwise comparisons with Tukey’s correction for expression-type revealed that participants were less accurate when responding to NM (*prob* = 0.344) than when responding to LIT (*prob* = 0.979; *OR* = 88.68, *z* = 17.921, *p* < 0.001), CM (*prob* = 0.951; *OR* = 36.661, *z* = 14.865, *p* < 0.001), or UR (*prob* = 0.929; *OR* = 25.063, *z* = 13.499, *p* < 0.001). In addition, participants were more accurate responding to LIT than to CM (*OR* = 2.419, *z* = 3.463, *p* = 0.003) or UR (*OR* = 3.535, *z* = 4.998, *p* < 0.001), but similar between CM and UR (*p* = 0.408).

**FIGURE 1:**
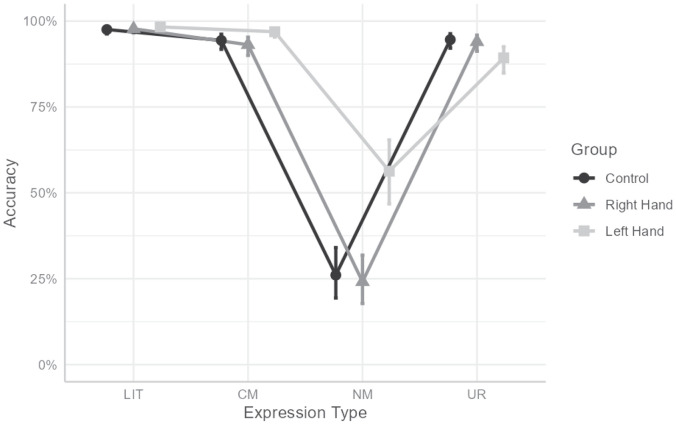
Interaction between hand and expression type for accuracy rate. LIT, literal expressions; CM, conventional metaphors; NM, novel metaphors; UR, unrelated expressions. Error bars indicate 95% CI.

More importantly, the interaction between hand and expression-type was found significant, χ^2^(6) = 396.674, *p* < 0.001 (see Figure 1). To understand the interaction, we computed omnibus tests conditionally for each expression-type, with Bonferroni’s correction, followed by a pairwise comparison between conditions with Tukey’s correction when simple effect were significant. This revealed a significant simple effect in NM, χ^2^(2) = 106.014, *p* < 0.001, indicating that participants in the left-hand contraction condition (*prob* = 0.563) were more accurate responding to NM than participants in the right-hand (*prob* = 0.241; *OR* = 4.049, *z* = 9.168, *p* < 0.001) or control (*prob* = 0.261; *OR* = 3.663, *z* = 8.622, *p* < 0.001) conditions. No difference between right-hand and control was found (*p* = 0.776).

A similar, though smaller, effect was found for CM, χ^2^(2) = 23.7, *p* < 0.001, indicating that participants in the left-hand contraction condition (*prob* = 0.969) were more accurate responding to CM than participants in the right-hand (*prob* = 0.931; *OR* = 2.309, *z* = 4.733, *p* < 0.001) or control (*prob* = 0.944; *OR* = 1.866, *z* = 3.54, *p* < 0.001) conditions. No difference between right-hand and control was found (*p* = 0.41). For UR, an opposite effect was found, χ^2^(2) = 22.852, *p* < 0.001, indicating that participants in the left-hand condition (*prop* = 0.893) were less accurate at responding to UR than participants in the right-hand (*prob* = 0.94; *OR* = 2.1, *z* = 4.405, *p* < 0.001) or control (*prob* = 0.946; *OR* = 1.873, *z* = 3.706, *p* < 0.001) conditions. No differences between hand conditions were found for LIT (χ^2^(2) = 3.786, *p* = 0.603).

### Reaction Time

Single trial reaction times were first submitted to a log (base 10) transformation. Transformed reaction times were then modeled with a linear mixed model, with Satterthwaite degrees of freedom. The unconditional interclass correlation was estimated separately for Participants and Items. A moderate amount of variation was found between participant in their overall mean reaction times (*N* = 119; *ICC* = 0.213, χ^2^(1) = 6,079.485, *p* < 0.001), indicating that some participants tended to systematically react slower or faster than others. Additionally, a moderate amount of variation was found between items (*N* = 240; *ICC* = 0.151, χ^2^(1) = 3,824.753, *p* < 0.001), indicating that, as expected, some items tended to evoke slower or faster responses.

For the main analysis, hand (left, right, no-contraction) and expression-type (LIT, CM, NM or UR) and their interaction were modeled as fixed effects. The random effect structure included crossed random intercepts per participant and item, and random slopes of the expression-type effect per participant. To avoid a singular fit, the random slopes for the hand effect per item were omitted, as well as the covariances between participants’ random effects. We report here type 3 omnibus tests for the fixed effects, and approximated $\eta_{p}^{2}$ effect sizes (Friedman, 1982). Conditional means are back-transformed for the reader’s convenience.

The generic mixed-level equation, with all effects considered to be random, was:


$${\text{log}}_{10}\left({\text{RT}}_{i}\right)∼N\left(\mu ,\sigma^{2}\right)$$

$$\mu =\alpha_{j\left[i\right],k\left[i\right]}+\beta_{ck\left[i\right]}\times \mathrm{Expression}{\mathrm{Type}}_{c}$$

$$\alpha_{j}∼N\left(\mu_{\alpha_{j}},\sigma_{\alpha_{j}}^{2}\right),\mathrm{for}\mathrm{Item}j=1,\ldots ,J$$

$$\begin{array}{c} \left(\begin{array}{c} \alpha_{k}\\ \beta_{ck} \end{array}\right)∼N\left(\left(\begin{array}{cc} \gamma_{0}^{\alpha_{\text{k}}}+\gamma_{\text{g}}^{\alpha_{\text{k}}}\times {\text{Hand}}_{\text{g}} & \\ \gamma_{0}^{\beta_{\text{ck}}}+\gamma_{\text{g}}^{\beta_{\text{ck}}}\times {\text{Hand}}_{\text{g}} & \end{array}\right),\left(\begin{array}{cc} \sigma_{\alpha_{\text{k}}}^{2} & 0\\ 0 & \sigma_{\beta_{\text{ck}}}^{2} \end{array}\right)\right)\\ ,\mathrm{for}\mathrm{Participant}k=1,\ldots ,K \end{array}$$

The analysis revealed a significant main effect for expression-type condition, *F*(3,332.161) = 86.28, *p* < 0.001, $\eta_{p}^{2}=0.438$. A pairwise comparison between conditions (with Tukey’s correction) revealed that participants responded faster to LIT (*M* = 496) than to CM (*M* = 537; *t*(241) = 4.22, *p* < 0.001), NM (*M* = 676; *t*(302) = 14.431, *p* < 0.001), and UR (*M* = 633; *t*(304) = 10.45, *p* < 0.001). In addition, they responded faster to CM than to NM (*t*(307) = 10.632, *p* < 0.001) and UR (*t*(316) = 7.011, *p* < 0.001); there was no significant difference between UR and NM (*p* = 0.059). No main effect for hand was found (*F*(2,125.266) = 0.601, *p* = 0.549, $\eta_{p}^{2}=0.009$).

**FIGURE 2:**
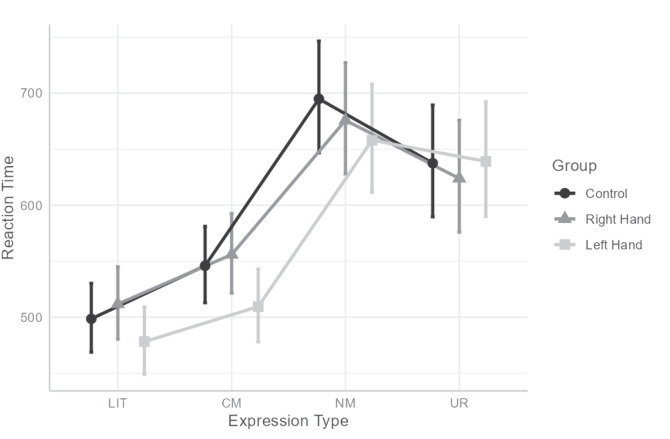
Interaction between hand and expression type for reaction time. LIT, literal expressions; CM, conventional metaphors; NM, novel metaphors; UR, unrelated expressions. Error bars indicate 95% CI.

Similarly to AC, an interaction between hand condition and expression type was found for RT, *F*(6,124.475) = 2.182, *p* = 0.049, $\eta_{p}^{2}=0.095$ (see Figure 2). To understand the interaction, we computed omnibus tests conditionally for each expression-type, with Bonferroni’s correction, followed by a pairwise comparison between conditions with Tukey’s correction when simple effect were significant. However, none of the omnibus tests were significant (*p*s > 0.399). Thus, an additional series of conditional omnibus tests was conducted, this time for the simple effect of expression type within each of the hand conditions. This analysis revealed that all three simple effects were significant: *F*(3,190.5) = 57.528, *p* < 0.001, $\eta_{p}^{2}=0.475$, for control; *F*(3,187.051) = 38.283, *p* < 0.001, $\eta_{p}^{2}=0.384$, for right- hand; and *F*(3,185.972) = 60.524, *p* < 0.001, $\eta_{p}^{2}=0.494$, for left-hand. Within all hand conditions, responses to LIT were faster than to CM, NM and UR and responses to CM were faster than to NM and UR (all *p*s < 0.014), but NM and UR did not differ (*p*s > 0.092).

## Discussion

The main objective of the present study was to find and test new, non-invasive, efficient, and simple methods to help enhance NM processing and understanding. This was tested through unilateral muscle contractions (i.e., ball squeezing), that is believed to cause a direct manipulation and activation of the contralateral hemisphere. Left-Hand contractions were expected to boost the RH coarse semantic processing, and thereby improve NM comprehension.

As anticipated, and in line with previous findings, this study found that the intentional activation of the RH, by contracting the left hand, resulted in better performance in a NM comprehension task. That is to say, participants who had squeezed the ball with their left-hand judged NM to be meaningful far better than those who had used their right-hand, or had not squeezed the ball at all (i.e., their accuracy rates were much higher, and they were more prone to judge NM as meaningful expressions). This, as far as we know, is the first time that NM comprehension has been shown to be improved in general, and in particular by such a simple and easy technique of a brief contraction of the left hand.

Moreover, this finding empirically adds to the support found in past studies for Beeman’s theory that the intact brain is characterized by LH lateralization for language processing, with RH becoming more involved when coarse semantic coding is required (Beeman et al., 1994). In his studies, Beeman et al. (1994), Beeman (2005) claimed that the RH is involved in language processing. The RH, by its nature, reflects a coarser language processing, which activates a larger (yet weaker) semantic field when exposed to a word in comparison with the LH, which activates a smaller, more focused, (yet stronger) semantic field. As a result of the larger semantic field activated by the RH, a unique semantic processing may occur. The large and diffuse semantic fields make the RH more sensitive to the overlap of different concepts that are activated by different words. Semantic overlap may be very valuable when individuals encounter language that requires linking between concepts that are seemingly unrelated, or only distantly so. Figurative language in general – and NM in particular – consists of concepts that are seemingly unrelated (or distantly related), and therefore require the special overlap that occurs in the RH. An individual with a weak or a damaged RH will have difficulty understanding figurative language. Our study has found that when the RH is intentionally activated, participants are indeed more accurate when deciding whether a given NM is meaningful or not. In other words, we have directly shown that in order to understand NM (i.e., coarse semantic coding), the RH is engaged.

Generally, it was found that participants who were in the left-hand contraction condition were more accurate than both control participants and right-hand contraction participants. It can be assumed that left-hand contractions improved the cooperation between the two hemispheres and, as a result, enhanced language processing in general. That is, less lateralization of the LH and more involvement of the RH resulted in enhanced language comprehension (Bartha-Doering et al., 2018). We hypothesize that, although there are hemispheric differences in language processing, the harmony between the two results in better comprehension of language in general (Beeman, 2005; Bartha-Doering et al., 2018).

As for CM, significant results indicated that participants who squeezed the ball with their left hand were more accurate in identifying them as meaningful expressions – but the effect size was much smaller than for NM. This small effect could be explained by the high familiarity of CM, which are well-known metaphors whose metaphoric meaning has become salient (Giora et al., 2000) – and given their high familiarity, coarse semantic coding (i.e., the RH) is less required. That is, the RH is involved in processing CM, but its involvement is smaller due to the familiarity of CM (Giora et al., 2000). It is important to note that our findings and references regarding CM are in relation to the neurotypical population alone and these findings cannot be attributed to other populations. For example, it was found that children with autistic spectrum disorders did not show better understanding of conventional metaphors than novel metaphors (Chahboun et al., 2016).

Conversely, the opposite effect was found for UR: participants who squeezed the ball with their left hand were significantly less accurate than in the other two conditions. The LH involvement in language provides us with the ability to distinguish between plausible and implausible semantic relationships. The LH is less sensitive to overlaps between distantly related concepts, so suppressing its activation may result in its inability to act as a gatekeeper that comes to our aid when encountering meaningless language expressions. For example, Zeev-Wolf et al. (2014) found that although individuals with schizophrenia were better at understanding NM due to their over-reliance on RH coarse semantic processing, they were unable to judge UR expressions: they more often found meaning in meaningless expressions. In our study, participants in the Left-Hand condition show a similar pattern – suggesting that boosting the activation of the RH, which improves NM comprehension, may come at the expense of accurate judgment of UR.

With regard to RT, it was found that RTs for NM were significantly slower than for other expression types. Participants did indeed find it harder to comprehend and process NM. This finding is in line with previous findings comparing NM to other types of stimuli (e.g., Mashal et al., 2005, 2007; Arzouan et al., 2007a, b). Moreover, the interaction between expression type and hand condition was also found to be significant. *Post hoc* analysis failed to explain the interaction, however, the pattern of descriptive results indicates that for right-hand contractions and Control participants, RT for NM was slower than the other expression types – but with Left-Hand contraction, RT for NM was only slower than LIT and CM and similar to UR (see Figure 2). This pattern mirrors the results found for AC – in that participants were more accurate in judging NM, but less accurate in judging UR.

Although the results in this study show that squeezing with the left-hand enhances RH activation, thereby improving coarse semantic processing, it should be noted that Turner et al.’s (2017) previous experiments did not yield the same pattern of results. In their study, they tried to replicate Goldstein et al. (2010) cognitive findings by means of the RAT (Mednick, 1968; Nevo and Levin, 1978) – a task involving both language and creativity. They found that, unlike in Goldstein et al. (2010) study, better scores on the RAT also were affected by right-hand contractions (i.e., activating the LH) – but not by left-hand ones. In other words, although it has been established that creativity, like figurative language, is supported by the mechanisms in the RH, Turner et al. (2017) found otherwise. To explain these different results, they postulated that different languages yield different results – to wit, English RAT problems are probably solved by verbal analytical processing (i.e., resulting from stimulating the LH) rather than creative insight (i.e., resulting from stimulating the RH). In other words, Turner et al. (2017) believe that the language selected matters; however, this assumption has not been tested directly. Moreover, the results of their second experiment (that included homographs interpretation improvement) showed that unilateral hand contractions did not, in fact, yield any significant effects. Turner et al. (2017) suggest that although previous studies have shown the effectiveness of unilateral hand contractions manipulation on emotion, motivation, and cognition, their extent and magnitude are limited to specific tasks. Thus, our study shows that unilateral hand contraction is efficient in improving coarse semantic processing.

Another possible explanation for the lack of consistent results may be that Turner et al. (2017) recruited participants that were both mixed-handers and strong right- handers. Previous studies recruited only strongly right-handed individuals, because they usually exhibit LH dominance in semantic processing, and RH activation when encountering figurative language (Rasmussen and Milner, 1977; Knecht et al., 2000; Li et al., 2015). Conversely, mixed-handers show greater access to the semantic networks in the RH, and therefore exhibit similar processing for closely and distantly related concepts (Rasmussen and Milner, 1977; Knecht et al., 2000; Sontam and Christman, 2012; Li et al., 2015).

As has been mentioned earlier, another study which examined differences in perception of human emotions through contraction conditions using photographs, yielded no significance results (Stanković and Nešić, 2020). According to Stanković and Nešić (2020), one explanation for this is the larger anatomical distance between the brain regions involved in visual emotional perception and the motor cortex (as opposed to a closer distance to the language or cognitive regions). That is, there is less brain activation sufficient for improved emotional (as opposed to cognitive) processing. They assume that emotional-visual perception is a complex task which might require the involvement of several brain regions; it is possible then, that the hemispheric activation of the motor areas seemed insufficient to activate and improve the emotional perception which rely on a more distributed brain region. In addition, we believe that the lack of sufficient power recruited for the experiment has also affected the results. Moreover, Stanković and Nešić (2020) used different measurements and procedures. In their experiment, they instructed the participants to squeeze a dynamometer (rather than a ball) and they did not mention if the squeezing was continual or intermittent. Lastly, Stanković and Nešić’s (2020) unilateral hand-contraction condition was much shorter than the one in this study. While in this study participants needed to contract the ball for 4 min, one time before the beginning of the experiment and two more during the breaks, in Stanković and Nešić’s (2020) experiment the contraction condition was much shorter and lasted only for 45 s before each block. In other words, the procedure of our experiment was longer and may have had a greater impact.

The present study has significant implications. First, its main contribution is highlighting the capability, and advantages, of boosting the involvement of the RH in language processing. As we have concluded, the RH’s unique performance relies on its ability to bring together distant associations, which the mechanisms in the LH do not provide. By demonstrating a boosting of RH performance, this study can help in enhancing the comprehension of figurative language among the neurotypical population. Furthermore, the method used in this study may provide a better understanding of new sophisticated figurative language expressions, as encountered in poetry, literature, or even in daily conversations.

Second, the RH’s access to non-dominant associations is proven to improve not only the understanding of figurative speech, but also of other non-verbal cognitive abilities, such as creative and divergent thinking, which also requires engagement of the RH (Goldstein et al., 2010). Thus, this study may also help certain target populations, that have low RH dominance, by improving their RH function and ability. Populations such as high-functioning individuals on the autistic spectrum disorder, exhibit RH dysfunctions that result in a weakened ability to link together pieces of information to make a whole idea. This dysfunction results in their inability to perform well on any task that requires comprehension or processing (Ozonoff and Miller, 1996). Through the intentional activation of the RH, these individuals will have an opportunity to improve their ability of understanding figurative language, to boost their creative thinking, and perhaps even their social skills, which rely in part on comprehension of figurative language (MacKay and Shaw, 2004).

Furthermore, psychological and paramedical services could use this study, and the method behind it, when dealing with language impairments and other disorders related to hemispherical asymmetries. It has been shown that comprehension of figurative language continues to develop rapidly in adolescence, and is completed only in adulthood (Patael et al., 2018). This is linked to the incomplete maturation of the RH during childhood and adolescence, and the consequent imperfect processing of figurative language (Patael et al., 2018). Thus, our study may assist teachers and researchers in the field of education and developmental psychology in helping children and adolescents to improve and develop their figurative language processing skills.

Lastly, our brains act like a muscle: they are flexible, and constantly change. For example, new synapses are constantly being created or destroyed (May, 2011; Zatorre et al., 2012; Lövdén et al., 2013). Although our findings demonstrate an immediate boost following the ball-squeezing (as opposed to a gradual improvement over time), they also highlight a new training method that may expand the processing ability of our brains to figurative language.

The current study has several limitations. First, our study was a behavioral one, that did not directly measure the engagement of each hemisphere. Further studies should validate that left-hand contraction does indeed improve NM processing, by activating brain regions in the RH that are involved in NM processing – e.g., by means of magnetoencephalograms, or fMRI. In addition, as previously noted by Turner et al. (2017), the input language used for assessment is probably also a factor. In other words, although we indeed found significant results in our study, further studies should check for RH activation and engagement (through hand contractions) in NM processing in various languages. Lastly, as we found, improving figurative language comprehension using left-hand contraction may come at the price of also accepting unrelated expressions as being meaningful. Notwithstanding these limitations, the findings of this study show that through motor activities, hemispheric balance can be artificially shifted toward greater activation of one hemisphere, which can greatly improve cognitive processing.

In conclusion, the purpose of the present work was to investigate figurative language improvement by means of a physiological method that manipulates our brains. We have shown that this method – which has already been found effective in other studies – can also be used to improve figurative language comprehension among adults. Figurative language – a fundamental aspect of our communication skills – may now be easily enhanced by means of a simple method.

## Data Availability Statement

The raw data supporting the conclusions of this article will be made available by the authors, without undue reservation.

## Ethics Statement

The studies involving human participants were reviewed and approved by the Ben-Gurion University HSR Committee. The patients/participants provided their written informed consent to participate in this study.

## Author Contributions

TN designed the methodology, run the study, and wrote and revised the manuscript. MZ-W conceptualized the idea, analyzed the data, and wrote and revised the manuscript. Both authors contributed to the article and approved the submitted version.

## Conflict of Interest

The authors declare that the research was conducted in the absence of any commercial or financial relationships that could be construed as a potential conflict of interest.

## Publisher’s Note

All claims expressed in this article are solely those of the authors and do not necessarily represent those of their affiliated organizations, or those of the publisher, the editors and the reviewers. Any product that may be evaluated in this article, or claim that may be made by its manufacturer, is not guaranteed or endorsed by the publisher.

## References

[B1] Arzouan Y. Goldstein A. Faust M. (2007a). Brainwaves are stethoscopes: ERP correlates of novel metaphor comprehension. *Brain Res.* 1160 69–81. 10.1016/j.brainres.2007.05.034 17597591

[B2] Arzouan Y. Goldstein A. Faust M. (2007b). Dynamics of hemispheric activity during metaphor comprehension: electrophysiological measures. *Neuroimage* 36 222–231. 10.1016/j.neuroimage.2007.02.015 17428685

[B3] Bartha-Doering L. Kollndorfer K. Kasprian G. Novak A. Schuler A. L. Fischmeister F. P. S. (2018). Weaker semantic language lateralization associated with better semantic language performance in healthy right-handed children. *Brain Behav.* 8:e01072. 10.1002/brb3.1072 30298640 PMC6236252

[B4] Bassel C. Schiff B. B. (2001). Unilateral vibrotactile stimulation induces emotional biases in cognition and performance. *Neuropsychologia* 39 282–287. 10.1016/s0028-3932(00)00116-0 11163606

[B5] Beeman M. (1993). Semantic processing in the right hemisphere may contribute to drawing inferences from discourse. *Brain Lang.* 44 80–120. 10.1006/brln.1993.1006 8467379

[B6] Beeman M. (2005). Bilateral brain processes for comprehending natural language. *Trends Cogn. Sci.* 9 512–518. 10.1016/j.tics.2005.09.009 16214387

[B7] Beeman M. Friedman R. B. Grafman J. Perez E. Diamond S. Lindsay M. B. (1994). Summation priming and coarse semantic coding in the right hemisphere. *J. Cogn. Neurosci.* 6 26–45. 10.1162/jocn.1994.6.1.26 23962328

[B8] Bookheimer S. (2002). Functional MRI of language: new approaches to understanding the cortical organization of semantic processing. *Annu. Rev. Neurosci.* 25 151–188. 10.1146/annurev.neuro.25.112701.142946 12052907

[B9] Bottini G. Corcoran R. Sterzi R. Paulesu E. Schenone P. Scarpa P. (1994). The role of the right hemisphere in the interpretation of figurative aspects of language. A positron emission tomography activation study. *Brain* 117 1241–1253. 10.1093/brain/117.6.1241 7820563

[B10] Chahboun S. Vulchanov V. Saldaña D. Eshuis H. Vulchanova M. (2016). Can you play with fire and not hurt yourself? A comparative study in figurative language comprehension between individuals with and without autism spectrum disorder. *PLoS One* 11:e0168571. 10.1371/journal.pone.0168571 28036344 PMC5201294

[B11] Christie B. R. Eadie B. D. Kannangara T. S. Robillard J. M. Shin J. Titterness A. K. (2008). Exercising our brains: how physical activity impacts synaptic plasticity in the dentate gyrus. *Neuromolecular Med.* 10 47–58. 10.1007/s12017-008-8033-2 18535925

[B12] Coulson S. Wu Y. C. (2005). Right hemisphere activation of joke-related information: an event-related brain potential study. *J. Cogn. Neurosci.* 17 494–506. 10.1162/0898929053279568 15814008

[B13] Crow T. J. (1997). Schizophrenia as failure of hemispheric dominance for language. *Trends Neurosci.* 20 339–343. 9246721 10.1016/s0166-2236(97)01071-0

[B14] Démonet J. F. Thierry G. Cardebat D. (2005). Renewal of the neurophysiology of language: functional neuroimaging. *Physiol. Rev.* 85 49–95. 10.1152/physrev.00049.2003 15618478

[B15] Erdfelder E. Faul F. Buchner A. (1996). GPOWER: a general power analysis program. *Behav. Res. Methods Instrum. Comput.* 28 1–11. 10.3758/bf03203630

[B16] Faust M. (2012). “Thinking outside the left box: the role of the right hemisphere in novel metaphor comprehension,” in *Advances in the Neural Substrates of Language: Toward A Synthesis of Basic Science and Clinical Research*, ed. Faust M. (Malden, MA: Wiley Blackwell), 425–448. 10.1002/9781118432501.ch21

[B17] Faust M. Mashal N. (2007). The role of the right cerebral hemisphere in processing novel metaphoric expressions taken from poetry: a divided visual field study. *Neuropsychologia* 45 860–870. 10.1016/j.neuropsychologia.2006.08.010 17010392

[B18] Fiore S. M. Schooler J. W. (1998). “Right hemisphere contributions to creative problem solving: converging evidence for divergent thinking,” in *Right Hemisphere Language Comprehension: Perspectives from Cognitive Neuroscience*, eds Beeman M. Chiarello C. (Mahwah, NJ: Erlbaum), 349–371.

[B19] Friedman H. (1982). Simplified determinations of statistical power, magnitude of effect and research sample sizes. *Educ. Psychol. Meas.* 42 521–526. 10.1177/001316448204200214

[B20] Gibbs R. W. Jr. Leggitt J. S. Turner E. A. (2002). “What’s special about figurative language in emotional communication?,” in *The Verbal Communication of Emotions*, ed Fussell S. R. (Hove: Psychology Press), 133–158. 10.4324/9781410606341-13

[B21] Giora R. (2007). Is metaphor special? *Brain Lang.* 100 111–114. 10.1016/j.bandl.2006.08.001 16956657

[B22] Giora R. Zaidel E. Soroker N. Batori G. Kasher A. (2000). Differential effects of right- and left-hemisphere damage on understanding sarcasm and metaphor. *Metaphor Symb.* 15 63–83. 10.1080/10926488.2000.9678865

[B23] Gold R. Faust M. (2010). Right hemisphere dysfunction and metaphor comprehension in young adults with Asperger syndrome. *J. Autism Dev. Disord.* 40 800–811. 10.1007/s10803-009-0930-1 20054629

[B24] Goldstein A. Revivo K. Kreitler M. Metuki N. (2010). Unilateral muscle contractions enhance creative thinking. *Psychon. Bull. Rev.* 17 895–899. 10.3758/PBR.17.6.895 21169586

[B25] Gunter H. L. Ghaziuddin M. Ellis H. D. (2002). Asperger syndrome: tests of right hemisphere functioning and interhemispheric communication. *J. Autism Dev. Disord.* 32 263–281. 10.1023/A:1016326701439 12199132

[B26] Harmon-Jones E. (2006). Unilateral right-hand contractions cause contralateral alpha power suppression and approach motivational affective experience. *Psychophysiology* 43 598–603. 10.1111/j.1469-8986.2006.00465.x 17076816

[B27] Josse G. Tzourio-Mazoyer N. (2004). Hemispheric specialization for language. *Brain Res. Rev.* 44 1–12. 10.1016/j.brainresrev.2003.10.001 14739000

[B28] Kay J. Lesser R. Coltheart R. M. (1992). *Psycholinguistic Assessment of Language Performance in Aphasia.* Hove: Lawrence Erlbaum.

[B29] Knecht S. Dräger B. Deppe M. Bobe L. Lohmann H. Flöel A. (2000). Handedness and hemispheric language dominance in healthy humans. *Brain* 123 2512–2518. 10.1093/brain/123.12.2512 11099452

[B30] Li M. Chen H. Wang J. Liu F. Wang Y. Lu F. (2015). Increased cortical thickness and altered functional connectivity of the right superior temporal gyrus in left-handers. *Neuropsychologia* 67 27–34. 10.1016/j.neuropsychologia.2014.11.033 25438031

[B31] Londei A. D’Ausilio A. Basso D. Sestieri C. Gratta C. D. Romani G. L. (2010). Sensory-motor brain network connectivity for speech comprehension. *Human Brain Mapp.* 31 567–580. 10.1002/hbm.20888 19780042 PMC6870571

[B32] Lövdén M. Wenger E. Mårtensson J. Lindenberger U. Bäckman L. (2013). Structural brain plasticity in adult learning and development. *Neurosci. Biobehav. Rev.* 37 2296–2310. 10.1016/j.neubiorev.2013.02.014 23458777

[B33] MacKay G. Shaw A. (2004). A comparative study of figurative language in children with autistic spectrum disorders. *Child Lang. Teach. Ther.* 20 13–32. 10.1191/0265659004ct261oa

[B34] Mashal N. Faust M. Hendler T. (2005). The role of the right hemisphere in processing nonsalient metaphorical meanings: application of principal components analysis to fMRI data. *Neuropsychologia* 43 2084–2100. 10.1016/j.neuropsychologia.2005.03.019 16243053

[B35] Mashal N. Faust M. Hendler T. Jung-Beeman M. (2007). An fMRI investigation of the neural correlates underlying the processing of novel metaphoric expressions. *Brain Lang.* 100 115–126. 10.1016/j.bandl.2005.10.005 16290261

[B36] May A. (2011). Experience-dependent structural plasticity in the adult human brain. *Trends Cogn. Sci.* 15 475–482. 10.1016/j.tics.2011.08.002 21906988

[B37] Mednick S. A. (1968). The remote associates test. *J. Creat. Behav.* 2 213–214. 10.1002/j.2162-6057.1968.tb00104.x

[B38] Meister I. G. Wilson S. M. Deblieck C. Wu A. D. Iacoboni M. (2007). The essential role of premotor cortex in speech perception. *Curr. Biol.* 17 1692–1696. 10.1016/j.cub.2007.08.064 17900904 PMC5536895

[B39] Nevo B. Levin I. (1978). Remote associates test: assessment of creativity in Hebrew. *Megamot* 24 87–98.

[B40] Oldfield R. C. (1971). The assessment and analysis of handedness: the Edinburgh inventory. *Neuropsychologia* 9 97–113. 10.1016/0028-3932(71)90067-4 5146491

[B41] Ozonoff S. Miller J. N. (1996). An exploration of right-hemisphere contributions to the pragmatic impairments of autism. *Brain Lang.* 52 411–434. 10.1006/brln.1996.0022 8653388

[B42] Patael S. Z. Borodkin K. Faust M. (2018). Developmental changes in hemispheric processing of ambiguous words during adolescence. *J. Neurolinguistics* 47 50–58. 10.1016/j.jneuroling.2018.02.007

[B43] Pobric G. Mashal N. Faust M. Lavidor M. (2008). The role of the right cerebral hemisphere in processing novel metaphoric expressions: a transcranial magnetic stimulation study. *J. Cogn. Neurosci.* 20 170–181. 10.1162/jocn.2008.20005 17919080

[B44] Pulvermüller F. (2005). Brain mechanisms linking language and action. *Nat. Rev. Neurosci.* 6 576–582. 10.1038/nrn1706 15959465

[B45] R Core Team (2021). *R: A Language and Environment for Statistical Computing.* Vienna: R Foundation for Statistical Computing.

[B46] Rasmussen T. Milner B. (1977). The role of early left-brain injury in determining lateralization of cerebral speech functions. *Ann. N. Y. Acad. Sci.* 299 355–369. 10.1111/j.1749-6632.1977.tb41921.x 101116

[B47] Schiff B. B. Lamon M. (1994). Inducing emotion by unilateral contraction of hand muscles. *Cortex* 30 247–254. 10.1016/S0010-9452(13)80196-7 7924348

[B48] Schiff B. B. Guirguis M. Kenwood C. Herman C. P. (1998). Asymmetrical hemispheric activation and behavioral persistence: effects of unilateral muscle contractions. *Neuropsychology* 12 526–532. 10.1037/0894-4105.12.4.526 9805322

[B49] Sommer I. E. C. Ramsey N. F. Kahn R. S. (2001). Language lateralization in schizophrenia, an fMRI study. *Schizophr. Res.* 52 57–67. 10.1016/S0920-9964(00)00180-8 11595392

[B50] Sontam V. Christman S. D. (2012). Semantic organisation and handedness: mixed-handedness is associated with more diffuse activation of ambiguous word associates. *Laterality* 17 38–50. 10.1080/1357650X.2010.529450 21598173

[B51] Sotillo M. Carretié L. Hinojosa J. A. Tapia M. Mercado F. López-Martín S. (2004). Neural activity associated with metaphor comprehension: spatial analysis. *Neurosci. Lett.* 373 5–9. 10.1016/j.neulet.2004.09.071 15555767

[B52] Stanković M. Nešić M. (2020). No evidence of improved emotion perception through unilateral hand contraction. *Percept. Mot. Skills* 127 126–141. 10.1177/0031512519888080 31771447

[B53] Tréhout M. Leroux E. Delcroix N. Dollfus S. (2017). Relationships between corpus callosum and language lateralization in patients with schizophrenia and bipolar disorders. *Bipolar Disord.* 19 496–504. 10.1111/bdi.12526 28834020

[B54] Turner C. E. Hahn M. E. Kellogg R. T. (2017). Semantic processing in the left versus right cerebral hemispheres following unilateral hand contractions. *Laterality* 22 219–232. 10.1080/1357650X.2016.1154861 26947117

[B55] Wechsler D. (1997). *Wechsler Adult Intelligence Scale*, 3rd Edn. San Antonio, TX: Psychological Corporation. 10.1037/t49755-000

[B56] Xu J. Kemeny S. Park G. Frattali C. Braun A. (2005). Language in context: emergent features of word, sentence, and narrative comprehension. *Neuroimage* 25 1002–1015. 10.1016/j.neuroimage.2004.12.013 15809000

[B57] Zatorre R. J. Fields R. D. Johansen-Berg H. (2012). Plasticity in gray and white: neuroimaging changes in brain structure during learning. *Nat. Neurosci.* 15 528–536. 10.1038/nn.3045 22426254 PMC3660656

[B58] Zeev-Wolf M. Faust M. Levkovitz Y. Harpaz Y. Goldstein A. (2015). Magnetoencephalographic evidence of early right hemisphere overactivation during metaphor comprehension in schizophrenia. *Psychophysiology* 52 770–781. 10.1111/psyp.12408 25603893

[B59] Zeev-Wolf M. Goldstein A. Levkovitz Y. Faust M. (2014). Fine-coarse semantic processing in schizophrenia: a reversed pattern of hemispheric dominance. *Neuropsychologia* 56 119–128. 10.1016/j.neuropsychologia.2014.01.008 24462952

